# Differential cytotoxicity mechanisms of copper complexed with disulfiram for combination chemotherapy in human endometrial cancer cells

**DOI:** 10.3389/fonc.2026.1760903

**Published:** 2026-01-29

**Authors:** Peng-Sin Liu, Zih-Syuan Wu, Shih-Ming Huang

**Affiliations:** 1 Graduate Institute of Medical Sciences, College of Medicine, National Defense Medical University, Taipei, Taiwan; 2 Department of Nursing, Tri-Service General Hospital, National Defense Medical University, Taipei, Taiwan; 3 Graduate Institute of Biochemistry, College of Biomedical Sciences, National Defense Medical University, Taipei, Taiwan

**Keywords:** cellular stress, cisplatin, combination chemotherapy, disulfiram, doxorubicin

## Abstract

**Introduction:**

Endometrial cancer has the highest incidence among gynecologic malignancies, with a global prevalence of 10–20%. Type II tumors are generally high-grade and recurrent. Combination chemotherapy can provide synergistic effects to combat drug resistance. The potential of using disulfiram (DSF) and copper (I or II) complexes for combination therapy remains unclear.

**Methods:**

The cytotoxic effects of DSF, copper (I or II), and the DSF/copper complex were evaluated in two human endometrial cancer cell lines, RL95–2 and HEC-1-A, using cell viability analysis, combination index analysis, flow cytometry, immunocytochemical methods, and western blotting.

**Results:**

CuCl_2_, unlike CuCl, acted synergistically with DSF to induce cytotoxicity in HEC-1-A cells, while both CuCl_2_ and CuCl showed synergy with DSF in RL95–2 cells. The DSF–Cu^+^/Cu^2+^ complexes induced apoptosis, lipid peroxidation, autophagy, DNA damage, and ER stress in both cell lines. The complexes increased cytosolic and mitochondrial ROS in HEC-1-A but not in RL95–2 cells. The DSF/Cu^+^ complex, but not DSF/Cu^2+^, caused mitochondrial depolarization in both lines. In combination with cisplatin or doxorubicin, only the DSF/Cu^+^ complex synergized with doxorubicin in RL95–2 cells. Except for CuCl with cisplatin or doxorubicin in RL95-2, DSF, CuCl, and CuCl_2_ synergized with these drugs in both cell lines.

**Discussion:**

These findings indicate differential effects of CuCl and CuCl_2_ when complexed with DSF in human endometrial cancer cells. Given the pressing need for innovative approaches to tackle endometrial cancer, we believe our findings contribute valuable insights into the molecular interactions and therapeutic potentials of DSF/copper complexes.

## 1 Introduction

Endometrial cancer has the highest incidence among gynecologic malignancies, with a global prevalence estimated at 10–20% (1). Typically, the lesions are confined to the uterus, and the standard treatment involves primary hysterectomy and bilateral salpingo-oophorectomy, along with adjuvant radiotherapy and chemotherapy for patients with risk factors (2). The most common tumors (type I) are usually low-grade, endometrioid-type, and estrogen-dependent tumors and are often associated with an excellent prognosis. In contrast, type II tumors, including serous, undifferentiated, and clear cell types, are generally high-grade tumors and have a tendency to recur, even when treated early (3). Precision in chemotherapy is crucial to prevent severe complications, particularly for individuals with type II endometrial cancer requiring prolonged salvage chemotherapy for cancer recurrence. In clinical practice, it has been suggested that the significance of combination chemotherapy lies in its potential to achieve synergistic effects and minimize drug doses for enhanced treatment specificity in these patients or to overcome chemotherapeutic drug resistance (4).

Reactive oxygen species (ROS) encompass hydrogen peroxide (H_2_O_2_), the superoxide anion (O_2_^.^), and the hydroxyl radical (OH^.^) (5, 6). The major endogenous sources of ROS include the mitochondria respiratory chain, NADPH oxidase, and peroxisomes (7). In recent years, extensive research has focused on the role of oxidative stress, which is characterized by an imbalance between ROS and antioxidants, in the pathophysiology of malignant transformation in endometriosis (8–11). However, the oxidant/antioxidant imbalance has dual effects as it can promote both carcinogenesis and cancer cell death. A moderate increase in ROS levels favors cell proliferation and survival. Yet, when ROS levels surpass a certain threshold, they may exceed the cell’s antioxidant capacity and induce cell death by oxidizing cellular macromolecules, such as lipids, nucleic acids, and proteins (12). Mounting evidence indicates that cancer cells demonstrate elevated intrinsic ROS stress due to metabolic abnormalities and oncogenic signaling [6]. Increased ROS levels activate redox signaling, which is essential for survival, and promote tumorigenesis; however, cancer cells also uphold an active antioxidant defense system to shield themselves from ROS-induced cell death (13). Interestingly, the current chemotherapeutic drugs induce higher ROS stress to surpass the anti-oxidative capacity of cancer cells, thereby leading to cell death (14, 15).

Copper (Cu^+^/Cu^2+^) homeostasis in cancers is increasingly recognized as an appealing target for anti-cancer drug development (16, 17). Numerous studies have suggested that the levels of copper in both serum and tumor tissues are significantly elevated in cancer patients compared to healthy individuals. Two primary approaches have been tested in both preclinical and clinical settings, including the use of copper chelators to reduce the bioavailability of copper and copper ionophores to increase intracellular copper levels to maintain (decrease via chelators or increase via transporters) the intracellular level of copper (18). The Fenton reaction, catalyzed by copper as well as iron (Fe^2+^/Fe^3+^), produces reactive hydroxyl radicals, leading to significant oxidative damage (19). Elevated oxidative stress results in protein oxidation, releasing copper ions from proteins and reducing levels of glutathione-bound Cu^+^. Cu^2+^ is then reduced by cellular reductants such as superoxide (O^2-^) and hydro-ascorbate to complete the catalytic cycle (20). Excessive copper ions may displace other metals from their native ligands in crucial enzymes, leading to impaired enzymatic activities, including specific prolyl hydroxylases that are responsible for the degradation of hypoxia-inducible factor-1 alpha protein (21). The binding of Cu^+^ to the MEK-1 protein stimulates the MEK1-dependent phosphorylation of ERK1/2, ultimately promoting tumor proliferation through the MAPK pathway. The role of copper, particularly its function in human endometrial cancer cells, warrants further investigation.

Disulfiram (DSF) has been used for about 70 years as the most well-known irreversible inhibitor of aldehyde dehydrogenase (ALDH) (22, 23). Beyond ALDH inhibition, DSF has attracted attention as a drug-repurposing candidate in oncology because its anticancer activity can be markedly potentiated by copper. DSF is rapidly converted to diethyldithiocarbamate (DDC), which chelates copper to form bioactive copper–dithiocarbamate complexes (e.g., Cu(DDC)_2_/CuET) (24). DSF/Cu has been reported to act as a copper ionophore that elevates intracellular copper and triggers copper-dependent oxidative stress, thereby pushing cancer cells beyond their antioxidant capacity (25). In parallel, DSF-derived CuET has been shown to disrupt proteostasis by targeting components of the p97/VCP segregase pathway, including binding to NPL4, leading to proteotoxic stress and cancer cell death (26). Consistent with a copper-dependent anticancer mode of action, DSF complexed with Cu^2+^ is generally more cytotoxic than DSF alone, and DSF complexes with other metals have been reported to lack comparable cytotoxicity, suggesting copper specificity. Importantly, several studies suggest that DSF/Cu may exhibit context-dependent tumor selectivity. For example, clonogenic survival assays showed that DSF (50–150 nM) combined with physiological copper levels (15 μM CuSO_4_) was selectively toxic to H292 NSCLC cells compared with normal human bronchial epithelial cells (27). In breast models, DSF–Cu was reported to potently inhibit proteasomal activity in cancer cells but not in normal immortalized MCF10A cells prior to apoptosis induction (28). DSF shows promise as a drug for cancer treatment due to its easy availability, cost-effectiveness, and lower adverse effects compared to traditional drugs. Another important characteristic of DSF is its potential to serve as a promising approach to proteasome inhibition. Additionally, DSF can suppress various cancer-associated pathways, including ROS, PIK, MAPK, NF-κB, ALDH, EGFR/Src/VEGF, and others (29–31). The current therapeutic use of DSF involves its role as a metal chelator that is primarily complexed with Cu^2+^ (32). Complexes of DSF with Fe^2+^, Co^2+^, Mg^2+^, Ni^2+^, or Mn^2+^ did not suppress cell viability as DSF/Cu^2+^ did, indicating that the cytotoxic effect of the DSF/Cu complex seems to be copper-specific (33). However, the effects of DSF complexes with Cu^+^ need to be investigated in human endometrial cancer cells.

Cisplatin is a widely used chemotherapeutic drug that functions by crosslinking with the N^7^ of guanine in DNA and inducing ROS, leading to cell death (34). Although cisplatin is effective as a cytotoxic drug for RL95–2 cells, it does not show the same effectiveness for HEC-1-A cells, regardless of the p53 status (35). This suggests the presence of an alternative pathway to cell death in p53-negative cells which is potentially associated with levels of estrogen receptor alpha and the status of PTEN. Managing type II or recurrent endometrial cancer presents a challenge due to the lack of an effective chemotherapy regimen (36). The current approach for cisplatin-refractory and cisplatin-resistant tumors involves using other monotherapies, such as gemcitabine, topotecan, liposomal doxorubicin with or without trabectedin, or paclitaxel (37, 38). Given the involvement of various genes and signaling pathways in the pathogenesis of endometrial cancer, targeting ROS appears to be a rational therapeutic approach. The repurposing of the DSF/copper complex as a therapeutic strategy could potentially help to overcome chemotherapy resistance during cancer therapy.

In this study, we administered the DSF/copper complex to type I and type II endometrial cancer cells to investigate the cytotoxic effects of Cu^+^, Cu^2+^, DSF, and their complexes with Cu^+^ or Cu^2+^ and to determine whether they acted synergistically with chemotherapeutic drugs such as cisplatin and doxorubicin. Our work not only elucidates the mechanisms underlying the cytotoxicity of these compounds and their complexes but also highlights the potential application of this complex in current chemotherapy treatments for endometrial cancer patients.

## 2 Materials and methods

### 2.1 Cell culture and chemicals

Human endometrial carcinoma RL95-2 (ATCC^®^CRL-1671^™^) and HEC-1-A (ATCC^®^HTB-112^™^) cell lines were purchased from the American Type Culture Collection (ATCC; Manassas, VA, USA). The RL95–2 cells were cultivated in Dulbecco’s Modified Eagle’s Medium Nutrient Mixture F-12 (DMEM/F12). The HEC-1-A cells were cultivated in McCoy’s 5A medium supplemented with 10% fetal bovine serum (FBS) and 1% penicillin–streptomycin (Thermo Fisher Scientific, Waltham, MA, USA). *N*-acetyl cysteine (NAC), copper (II) chloride (CuCl_2_), 2′,7-dichlorofluorescein diacetate (DCFH-DA), disulfiram (DSF), Erastin, hydrogen peroxide (H_2_O_2_), propidium iodide (PI), and thiazolyl blue tetrazlium bromide (MTT) were obtained from Sigma Aldrich (St. Louis, MO, USA). Copper (I) chloride (CuCl) was obtained from Alfa Aesar (Tewksbury, MA, USA).

### 2.2 Cell survival analysis

The RL95-2 (8x10^3^) and HEC-1-A (5 × 10^3^) cells were plated in 96-well plates and cultured in the presence of the indicated drugs. The cells were then incubated with MTT solution (0.5 mg/mL in PBS) for 1 h at 37°C, after which dimethyl sulfoxide (100 μl) was added, and the absorbances at 570 nm and 650 nm were measured using a plate reader (Multiskan EX, Thermo, Waltham, MA, USA). The combination index (CI) was calculated using CalcuSyn (Biosoft, Cambridge, UK) to generate the isobologram as previously described (39). In general, CI < 1 indicates a synergistic combination effect, and CI > 1 indicates an antagonistic combination effect (40).

### 2.3 Apoptosis, cell cycle profiles, cytosolic/mitochondrial ROS, lipid peroxidation, and autophagy analyses

Early- and late-stage apoptotic cells were evaluated using a fluorescein phycoerythrin (PE) Annexin V Apoptosis Detection Kit (BD Biosciences, Franklin Lakes, NJ, USA) according to the manufacturer’s protocol. RL95-2 (6x10^5^) and HEC-1-A (3x10^5^) cells were plated in 6-well plates and cultured in the presence of either DSF, CuCl, CuCl_2_, or a combination of DSF with CuCl or CuCl_2_. The cells were stained with 5 μl PE Annexin V and 5 μl 7-amino-actinomycin (7-AAD) (5 μg/mL), which enabled identification of the early apoptotic cells. The viable cells were PE Annexin V- and 7-AAD-negative; the early apoptotic cells were PE Annexin V-positive and 7-AAD-negative; and the late apoptotic and dead cells were both PE Annexin V- and 7-AAD-positive.

The cell cycle profiles were evaluated based on cellular DNA content using FACSCalibur flow cytometry. RL95-2 (6x10^5^) and HEC-1-A (3x10^5^) cells were plated in 6-well plates and cultured in the presence of either DSF, CuCl, CuCl_2_, or a combination of DSF with CuCl or CuCl_2_. The cells were fixed in 70% ice-cold ethanol and stored at −30°C overnight, after which they were washed twice with ice-cold PBS supplemented with 1% FBS and stained with propidium iodide (PI) solution (5 μg/mL PI in PBS, 0.5% Triton x-100, and 0.5 μg/mL RNase A) for 30 min at 37 °C in the dark. The cell-cycle distribution was then evaluated using FACS, based on cellular DNA content.

The fluorescent markers DCFH-DA and MitoSOX™ Red (Invitrogen, Carlsbad, CA, USA) were used to determine the intracellular and mitochondrial ROS levels, respectively. RL95-2 (6x10^5^) and HEC-1-A (3x10^5^) cells were plated in 6-well plates and cultured in the presence of either DSF, CuCl, CuCl_2_, or a combination of DSF with CuCl or CuCl_2_. The cells were incubated for the indicated times with different combinations of DSF–Cu^+^/Cu^2+^ complexes. The living cells were then stained with DCFH-DA (10 μM) and MitoSOX™ (5 μM) Red for 30 min at 37 °C and harvested. After washing the cells once with PBS, they were evaluated using a FACSCalibur flow cytometer and Cell Quest Pro software (BD Biosciences, Franklin Lakes, NJ, USA).

The lipid peroxidation levels were measured using C11-BODIPY dye (D3861, ThermoFisher Scientific, Waltham, MA, USA) with flow cytometry, following the manufacturer’s instructions. RL95-2 (6x10^5^) and HEC-1-A (3x10^5^) cells were plated in 6-well plates and cultured in the presence of either DSF, CuCl, CuCl_2_, or a combination of DSF with CuCl or CuCl_2_. The cells were seeded and treated with the indicated drug in six-well plates for the indicated time. Subsequently, the medium was replaced with 10 µM C11-BODIPY-containing medium for 1 hour. After incubation, the cells were harvested using trypsin, washed three times with ice-cold PBS, and then re-suspended in PBS with 1% BSA. The lipid peroxidation levels within the cells were assessed using a FACSCalibur flow cytometer and Cell Quest Pro software.

The autophagy flux was measured using DALGreen (D675, Dojindo Molecular Technologies, Kumamoto, Japan). RL95-2 (6x10^5^) and HEC-1-A (3x10^5^) cells were plated in 6-well plates and cultured in the presence of either DSF, CuCl, CuCl_2_, or a combination of DSF with CuCl or CuCl_2_. After drug treatment, the cells were washed three times with PBS and then incubated with 1 μM DALGreen in the culture medium for 1 hour at 37°C. After washing three times with PBS, the fluorescence intensity was evaluated using a FACSCalibur flow cytometer and Cell Quest Pro software.

### 2.4 Immunocytochemistry

Immunocytochemical analysis was performed with RL95-2 (1.5x10^5^) and HEC-1-A (8x10^4^) cells adhered to cover slips in 24-well plates. After treatment with the indicated drug, the cells were fixed for 10 min in 4% formaldehyde, incubated for 10 min in 0.1% Triton X-100 solution, washed 3 times in PBS, and treated for 1 h at room temperature with 1% BSA. Thereafter, the cells were incubated with anti-TOM20 antibody at 4°C overnight. The next day, the cells were washed with PBS and incubated for 1 h with FITC-conjugated secondary antibody. The cell nuclei were stained with DAPI. The mitochondrial morphology was observed using a THUNDER Imager microscope equipped with a 100× objective (Leica, Wetzlar, Germany).

### 2.5 Mitochondrial membrane potential analysis

The RL95-2 (6x10^5^) and HEC-1-A (3x10^5^) cells were seeded into 6-well plates and cultured in the presence of either DSF, CuCl, CuCl_2_, or a combination of DSF with CuCl or CuCl_2_. The following day, the cells were treated with the indicated drugs in fresh medium for the specified durations. After treatment, both the dead and viable cells were collected, washed with PBS, and incubated with 1× binding buffer containing the MMP-sensitive fluorescent dye JC-1 for 30 min at 37°C in the dark. Subsequently, the cells were then washed twice with PBS, resuspended in 500 μl of 1× binding buffer, and analyzed using a FACSCalibur flow cytometer and Cell Quest Pro software (BD Biosciences, Franklin Lakes, NJ, USA). The cell-volume gating strategy involved forward scatter height (FSC-H) and side scatter height (SSC-H), and the median fluorescence intensity of the vehicle was used as the starting point for M2 gating. Mitochondrial depolarization was determined from a decrease in the red/green fluorescence intensity ratio.

### 2.6 Western blotting

The drug-treated RL95-2 (6x10^5^) and HEC-1-A (3x10^5^) cells were lysed in RIPA buffer (100 mM Tris-HCl (pH 8.0), 150 mM NaCl, 0.1% SDS, and 1% Triton 100) at 4°C. The proteins in the resultant lysates were separated using 12% SDS-PAGE, transferring to PVDF membrane, blocked for 1 hour with 5% non-fat milk in 1x TBST, and incubating the membrane with primary antibodies (all at a dilution of 1:1000) at 4°C overnight. The primary antibodies included against mtTFA, PGC-1α, β-actin, p53, p21, CyclinB1, ATF3, p62 (Santa Cruz Biotechnology, Santa Cruz, CA, USA), CHOP, LC3BI/II, PARP (Cell Signaling, Danvers, MA, USA), cyclin D1, γH2AX, (Abcam, Cambridge, UK), and HO-1 (Enzo Life Sciences, Farmingdale, NY, USA). The blots were subsequently incubated with HRP-conjugated secondary antibodies (all at a dilution of 1:10000) (anti-mouse IgG, AP192P; and anti-rabbit IgG, AP132P, Merck-Millipore). The immunoreactive proteins were detected using the ECLTM Western Blotting Detection Reagent and Amersham Hyperfilm™ ECL (GE Healthcare, USA), as previously described (39).

### 2.7 Statistical analysis

The values are expressed as the mean ± SD of at least three independent experiments. All comparisons between groups were made using Student’s *t*-tests. Statistical significance was set at *p* < 0.05.

## 3 Results

### 3.1 Effects of copper, DSF, and DSF–Cu^+^/Cu^2+^ complexes on cell viability, apoptosis, and cell cycle profile in HEC-1-A and RL95–2 cells

**Figure 1:**
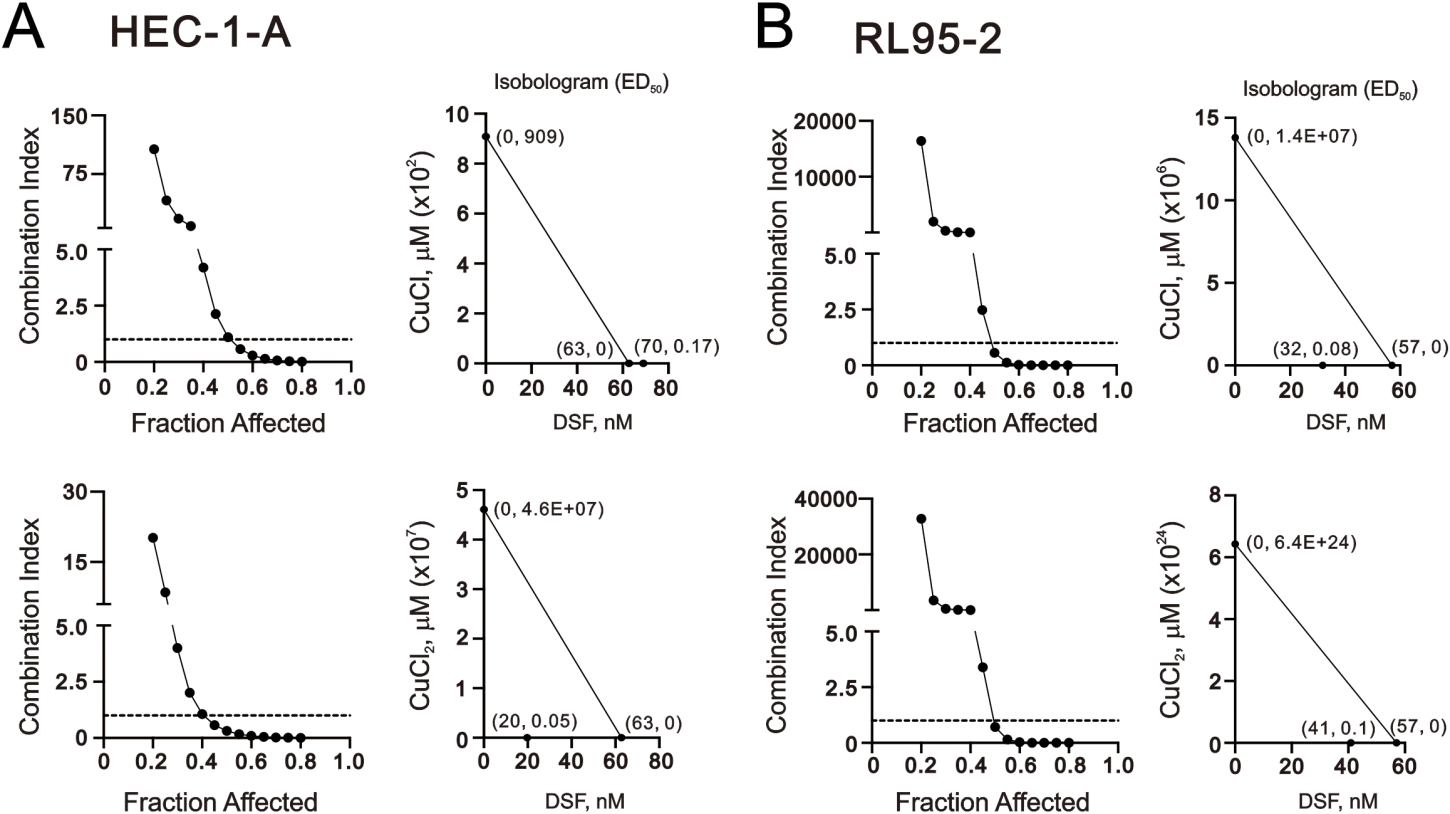
Combination index of DSF with CuCl and CuCl_2_ in HEC-1-A and RL95–2 cells. **(A, B)** HEC-1-A (3 × 10^5^) and RL95-2 (6 × 10^5^) cells were treated with DSF dose: 0, 0.0625, 0.125, 0.25, 0.5, 1, 2, and 4 µM combined with CuCl dose: 0, 0.03906, 0.07813, 0.15625, 0.3125, 0.625, 1.25, 2.5, 5, and 10 μM or CuCl_2_ dose: 0, 0.03906, 0.07813, 0.15625, 0.3125, 0.625, 1.25, 2.5, 5, and 10 μM for 24h. Cell viability was measured using the MTT method. Fraction affected values (indicating the fraction of cells inhibited after drug exposure) were obtained after exposure of the cells to a series of drug concentrations. Isobolograms were also constructed by plotting drugs concentrations (alone and in combination) that inhibits 50% cell viability. The combination index and Isobolograms (ED_50_) were calculated using CalcuSyn software.

The cytotoxicity of copper and the DSF–Cu^+^/Cu^2+^ complexes has previously been documented (25, 41, 42). It was necessary to investigate the cytotoxic effects of Cu^+^, Cu^2+^, DSF, or the combination of Cu^+^ or Cu^2+^ with DSF in HEC-1-A and RL95–2 cells. Isobologram analysis, which has been mathematically proven and widely used to evaluate drug interactions, was employed (43). This analysis assesses the dose at which two drugs, when used alone, have the same efficacy; this is generally expressed as the half-effective dose (ED_50_). We designed combinations of concentrations and calculated the combination index (CI) between CuCl (Cu^+^) or CuCl_2_ (Cu^2+^) with DSF in HEC-1-A and RL95–2 cells. We first calculated the value of ED_50_ for the treatment of CuCl, CuCl_2_, and DSF alone from the isobologram in HEC-1-A and RL95–2 cells (**Figures 1A, B**). The ED_50_ of DSF was 63 nM in the HEC-1-A cells and 57 nM in the RL95–2 cells. The ED_50_ of Cu^+^ was 909 µM in the HEC-1 cells and 1.4x10^7^ µM in the RL95–2 cells; the ED_50_ of Cu^2+^ was 4.6x10^7^ µM in the HEC-1 cells and 6.4x10^24^ µM in the RL95–2 cells, suggesting that Cu^+^ and Cu^2+^ both had no therapeutic effect on either of the cell lines. The definition of CI<1 as a synergistic effect was observed when the HEC-1-A and RL95–2 cells were treated with the Cu^2+^/DSF complex. The CI<1 of Cu^+^/DSF complex was measured in the RL95–2 cells, but not in the HEC-1-A cells. The combination of Cu^+^ and Cu^2+^ with DSF apparently decreased the ED_50_ of Cu^+^ (170 nM in the HEC-1-A cells and 80 nM in the RL95–2 cells) and Cu^2+^ (50 nM in the HEC-1-A cells and 100 nM in the RL95–2 cells), along with the decrease in the DSF’s ED_50_ from 63 nM to 20 nM (Cu^2+^) and from 57 nM to 32 nM (Cu^+^) and to 41 nM (Cu^2+^) in the HEC-1-A and RL95–2 cells, respectively.

**Figure 2:**
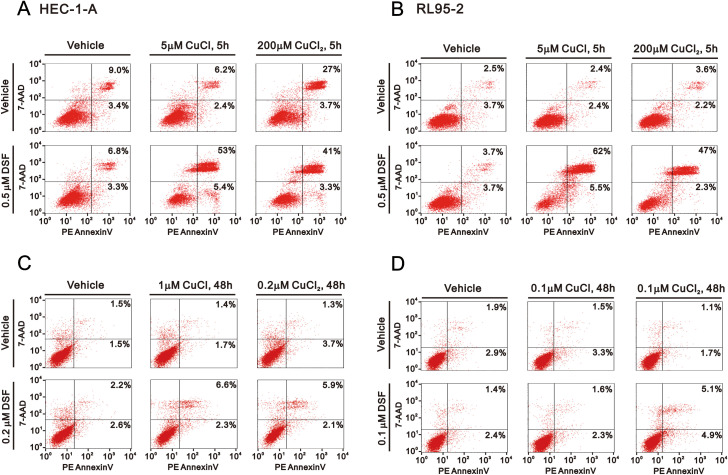
Effects of CuCl, CuCl_2_, and DSF on apoptosis in HEC-1-A and RL95–2 cells. **(A)** HEC-1-A and **(B)** RL95-2 (6 × 10^5^) cells were treated with 5 μM CuCl, 200 μM CuCl_2_, and 0.5 μM DSF for 5 h **(C)** HEC-1-A (3 × 10^5^) cells were treated with 1 μM CuCl, 0.2 μM CuCl_2_, and 0.2 μM DSF and **(D)** RL95–2 cells were treated with 0.1 μM CuCl, 0.1μM CuCl_2_, and 0.1 μM DSF for 48 h Cellular apoptosis was measured using the Annexin V apoptosis analysis with 7-AAD staining. Early apoptotic cells are PE Annexin V-positive and 7-AAD-negative, while late apoptotic cells are both PE Annexin V-positive and 7-AAD-positive. The results are representative of three independent experiments.

Elevated copper concentrations have been found to correlate with the cancer stage and/or progression in various types of tumors (44). The cytotoxicity of copper has been observed under both physiological and pathological conditions. For instance, the IC_50_ of copper sulfate was found to be 225–300 μM in HeLa cells (45). However, as demonstrated in **Figure 1**, Cu^+^ and Cu^2+^ had no discernible therapeutic effect in the HEC-1-A and RL95–2 cells. Several studies have supported the varying cytotoxic effects of different concentrations of DSF and Cu^+^/Cu^2+^ in cancer cells (33, 46). Hence, we examined the differential combinations of DSF and Cu^+^/Cu^2+^ using the Annexin V-apoptotic analysis (**Figure 2**). Firstly, we examined the induction of apoptosis with 0.5 μM DSF complexed with 5 μM Cu^+^ or 200 μM Cu^2+^ in the HEC-1-A and RL95–2 cells (**Figure 2**, top panels). With the exception of Cu^2+^ in the HEC-1-A cells, the total apoptotic populations were only induced by the combination of DSF and Cu^+^ or Cu^2+^ in both cell lines in the 5 h treatment. Next, the combinations of relatively lower concentrations of DSF and Cu^+^ or Cu^2+^ were applied in the Annexin V-apoptotic analysis (**Figure 2**, bottom panels). The induction of total apoptotic populations was observed in the HEC-1-A cells treated with 0.2 μM DSF complexed with 1 μM Cu^+^ or 0.2 μM Cu^2+^and the RL95–2 cells treated with 0.1 μM DSF complexed with 0.1 μM Cu^2+^ for 48 h.

**Figure 3:**
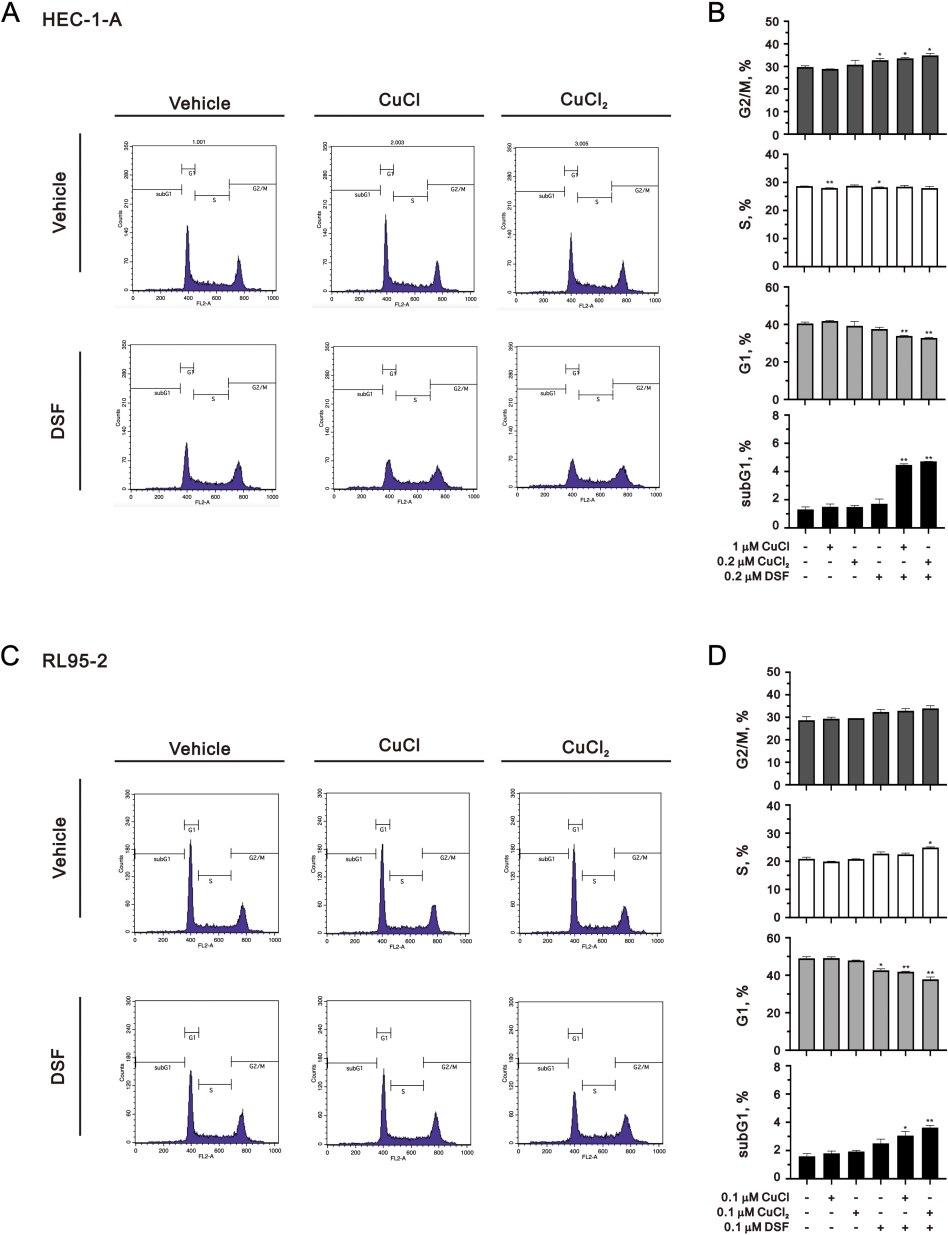
Effects of CuCl, CuCl_2_, and DSF on the cell cycle of human endometrial cancer cells. **(A, B)** HEC-1-A (3 × 10^5^) cells were treated with 1 μM CuCl, 0.2 μM CuCl_2_, and 0.2 μM DSF, and **(C, D)** RL95-2 (6 × 10^5^) cells were treated with 0.1 μM CuCl, 0.1 μM CuCl_2_, and 0.1 μM DSF for 48 h Cells were stained with propidium iodide (PI) and analyzed using flow cytometry. Bars depict the mean ± SD of three independent experiments. Student’s t-tests were analyzed and compared with vehicle. *p <0.05 and **p <0.01 (Student’s t-tests).

To avoid overly stressful cellular conditions, we further applied 0.2 μM DSF complexed with 1 μM Cu^+^ or 0.2 μM Cu^2+^ to the HEC-1-A cells and 0.1 μM DSF complexed with 0.1 μM Cu^2+^ to the RL95–2 cells for 48 h to address their effects on the cell cycle profile, cytosolic ROS, mitochondrial ROS, and lipid peroxide and ICC analysis. The PI cell cycle profiles were performed using flow cytometry analysis. Our data showed that the combination of DSF with Cu^+^ or Cu^2+^ increased the populations of the subG1 and G2/M phase and significantly decreased the population of the G1 phase in the HEC-1-A and RL95–2 cells (**Figure 3**).

### 3.2 Effects of copper, DSF, and DSF–Cu^+^/Cu^2+^ complexes on cytosolic ROS, mitochondrial ROS, lipid peroxidation, and autophagy in HEC-1-A and RL95–2 cells

**Figure 4:**
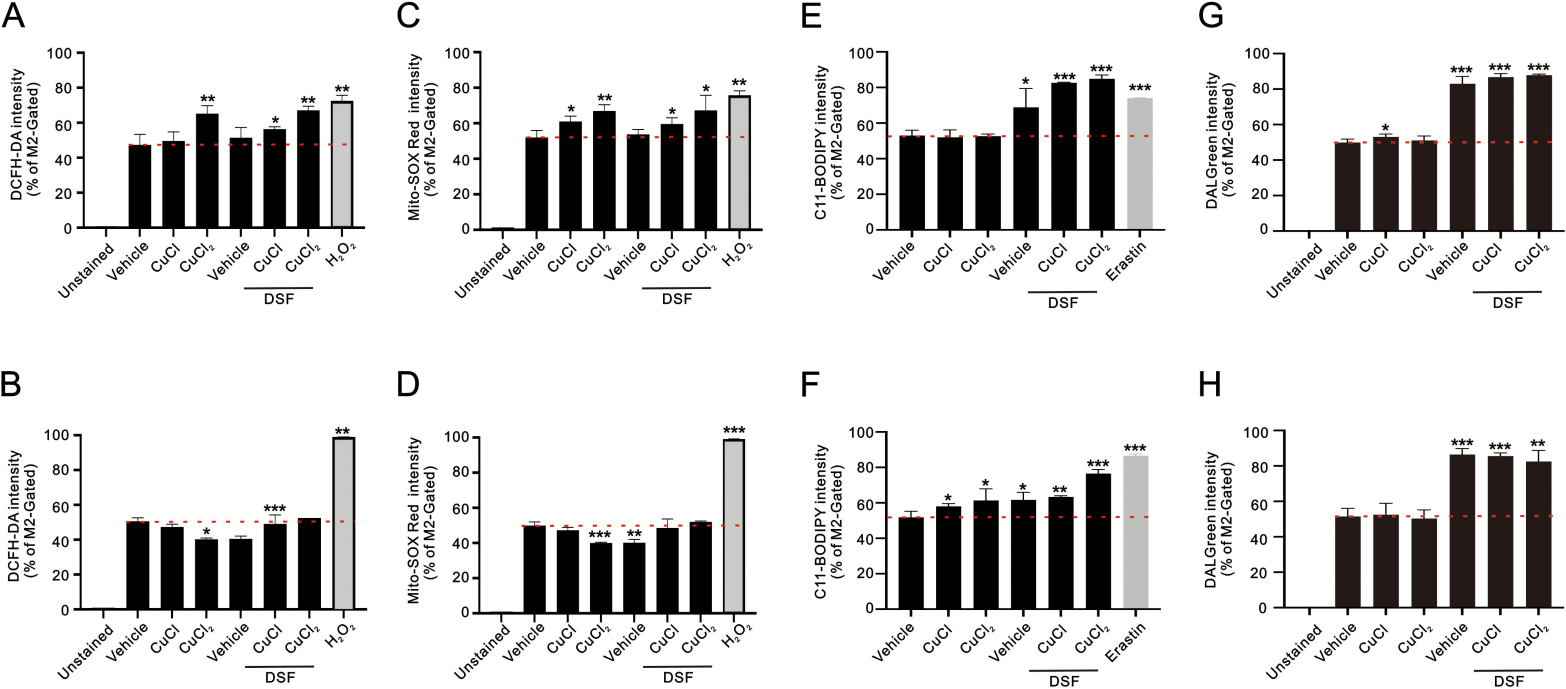
Effects of CuCl, CuCl_2_, and DSF on cytosolic and mitochondrial ROS levels, lipid peroxidation, and autophagy of human endometrial cancer cells. **(A-D)** HEC-1-A (3 × 10^5^) cells were treated with 1 μM CuCl, 0.2 μM CuCl_2_, and 0.2 μM DSF, and **(E-H)** RL95-2 (6 × 10^5^) cells were treated with 0.1 μM CuCl, 0.1 μM CuCl_2_, and 0.1 μM DSF. **(A, E)** Intracellular ROS and **(B, F)** mitochondrial ROS were assessed after 1.5 h of drug treatment using DCFH-DA and MitoSOX Red staining, respectively. **(C, G)** Lipid peroxidation and **(D, H)** autophagy were assessed after 48 h of treatment using C11-BODIPY and DALGreen staining, followed by flow cytometry analysis. Bars depict the mean ± SD of three independent experiments. Student’s t-tests were analyzed and compared with vehicle. *p <0.05, **p <0.01, and ***p <0.001 (Student’s t-tests). The red line was for each basal intensity.

We examined the effect of DSF–Cu^+^/Cu^2+^ complexes on cytosolic ROS using flow cytometry analysis with DCFH-DA dye in HEC-1-A and RL95–2 cells (**Figures 4A, E**). Compared with the hydrogen peroxide positive control, the Cu^2+^ and DSF–Cu^+^/Cu^2+^ complexes elevated cytosolic ROS in the HEC-1-A cells, and the Cu^+^, Cu^2+^, DSF, and DSF–Cu^+^/Cu^2+^ complexes decreased cytosolic ROS in the RL95–2 cells. We examined the effect of DSF–Cu^+^/Cu^2+^ complexes on mitochondrial ROS using MitoSOX dye in HEC-1-A and RL95–2 cells (**Figures 4B, F**). Compared with the hydrogen peroxide positive control, the Cu^+^, Cu^2+^, and DSF–Cu^+^/Cu^2+^ complexes significantly elevated mitochondrial ROS in the HEC-1-A cells, and the Cu^+^, Cu^2+^, DSF, and DSF–Cu^+^/Cu^2+^ complexes decreased cytosolic ROS in the RL95–2 cells. We further examined the effect of DSF–Cu^+^/Cu^2+^ complexes on the level of lipid peroxide using C-11 BODIPY dye in HEC-1-A and RL95–2 cells (**Figures 4C, G**). Compared with the Erastin positive control, the DSF and DSF–Cu^+^/Cu^2+^ complexes elevated the level of lipid peroxide in the HEC-1-A cells, and the Cu^+^, Cu^2+^, DSF, and DSF–Cu^+^/Cu^2+^ complexes all elevated the level of lipid peroxide in the RL95–2 cells. The fluorescence of DALGreen intensifies at acidic pH, making it suitable for monitoring the degradation phase of autophagy, specifically autophagolysosomes (**Figures 4D, H**). Our data showed that the DSF and DSF–Cu^+^/Cu^2+^ complexes induced the formation of autophagosomes in the HEC-1-A and RL95–2 cells. Cu^+^ was able to form autophagolysosomes in the HEC-1-A cells.

### 3.3 Effects of copper, DSF, and DSF–Cu^+^/Cu^2+^ complexes on mitochondrial dynamics in HEC-1-A and RL95–2 cells

**Figure 5:**
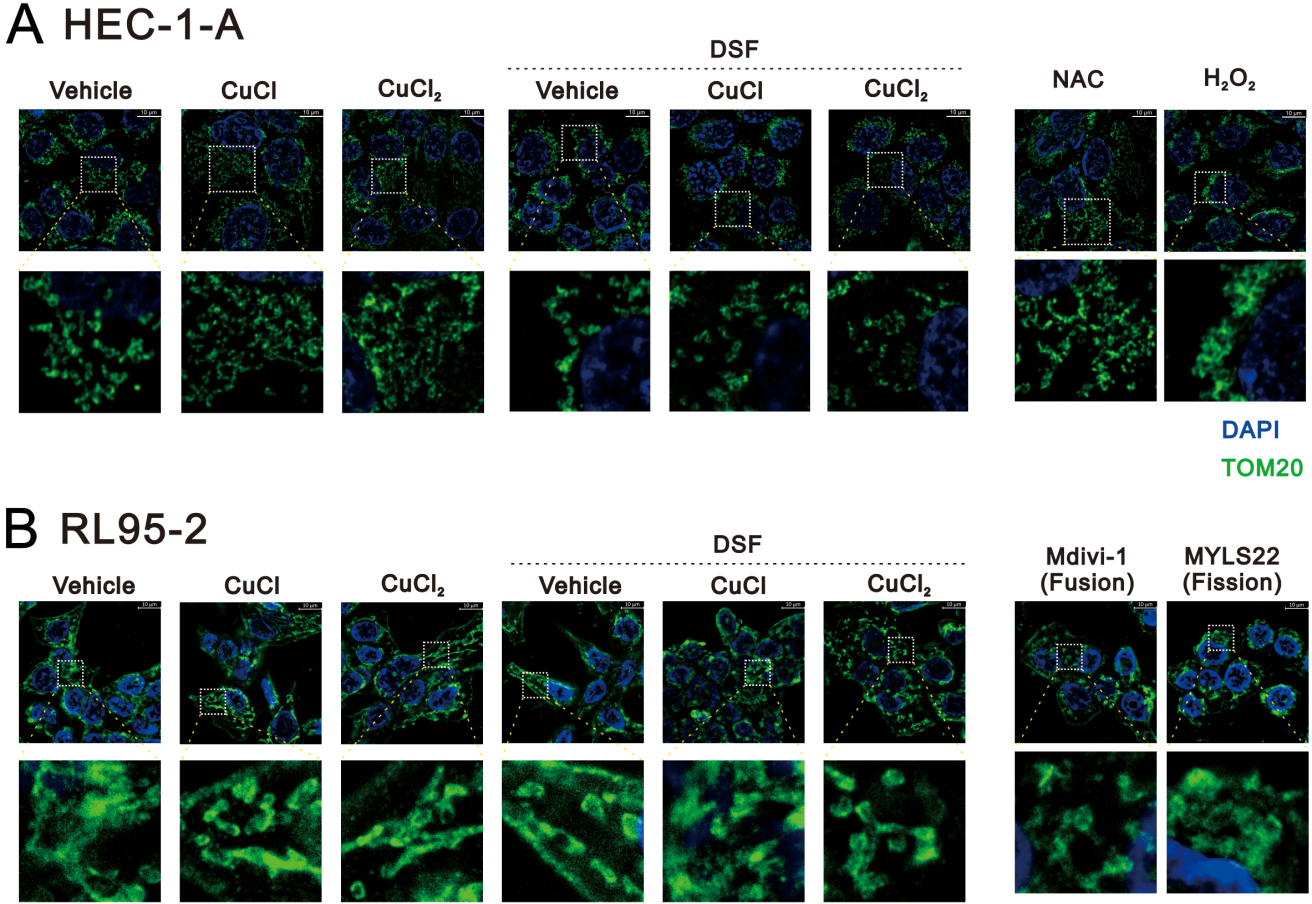
Effects of CuCl, CuCl_2_, and DSF on the mitochondrial morphology of human endometrial cancer cells. **(A)** HEC-1-A (8 × 10^4^) cells were treated with 1 μM CuCl, 0.2 μM CuCl_2_, and 0.2 μM DSF for 48h. NAC and hydrogen peroxide (H_2_O_2_) were the standard of reduced and oxidative status in HEC-1-A cells. **(B)** RL95-2 (1.5 × 10^5^) cells were treated with 0.1 μM CuCl, 0.1 μM CuCl_2_, and 0.1 μM DSF for 48 h Mdivi-1 and MYLS22 (20 μM) were the inducers for fusion and fission, respectively, of RL95–2 cells. Immunofluorescence labeling of the cells was performed with TOM20 (green) and DAPI (blue). Images were acquired with Leica Thunder microscope with a 100x objective. Scale bar=10 μm.

Furthermore, studies have found that mitochondrial dysfunction contributes to the resistance of these tumors to treatment (47–50). It has been demonstrated that mitochondrial dynamics and bioenergetic metabolism play a role in cisplatin resistance. ICC analysis was used to evaluate the effects of Cu^+^, Cu^2+^, DSF, and DSF–Cu^+^/Cu^2+^ complexes on the fusion–fission status of mitochondria in HEC-1-A and RL95–2 cells (**Figure 5**). In the HEC-1-A cells, we applied NAC (reduced status) and hydrogen peroxide (oxidized status) to monitor the mitochondrial dynamics via the mitochondrial outer membrane protein TOM20 (**Figure 5A**). The DSF and DSF–Cu^+^/Cu^2+^ complexes favored the oxidized status (fission status), and the others favored the reduced status (fusion status). In the RL95–2 cells, we applied fusion- and fission-specific drugs, Mdivi-1 (fusion status) and MYLS22 (fission status), to compare the effects of Cu^+^, Cu^2+^, DSF, and DSF–Cu^+^/Cu^2+^ complexes (**Figure 5B**). Our data showed that the DSF–Cu^+^/Cu^2+^ complexes favored the fusion status and that the others favored the fission status.

**Figure 6:**
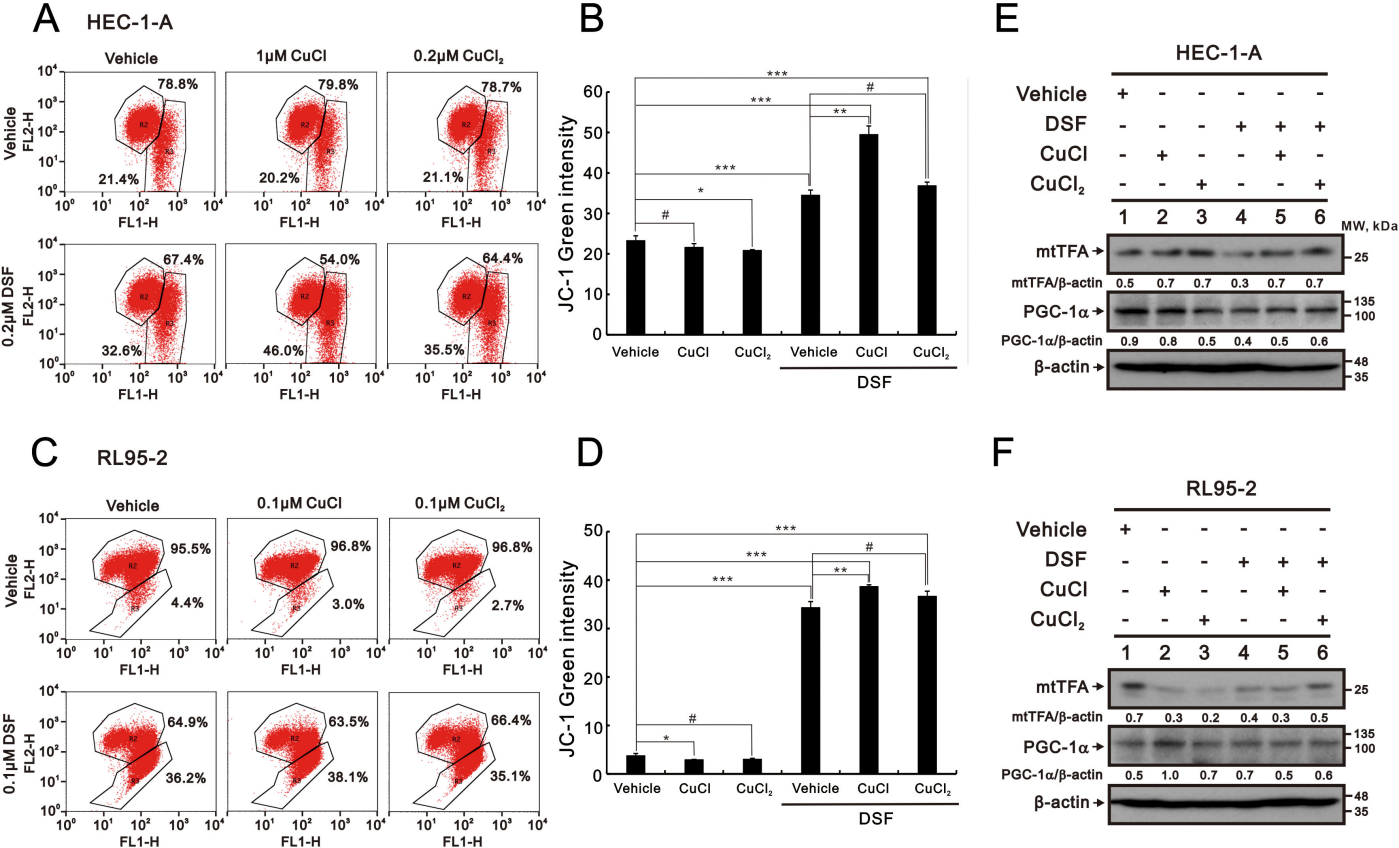
Effects of CuCl, CuCl_2_, and DSF on the mitochondrial membrane potential of human endometrial cancer cells. **(A, B)** HEC-1-A (3 × 10^5^) cells were treated with 1 μM CuCl, 0.2 μM CuCl_2_, and 0.2 μM DSF, and **(C, D)** RL95-2 (6 × 10^5^) cells were treated with 0.1 μM CuCl, 0.1 μM CuCl_2_, and 0.1 μM DSF for 48 h Mitochondrial membrane potential was assessed using JC-1 staining with flow cytometry. **(B, D)** The percentages of green fluorescence were plotted. Bars depict the mean ± SD of three independent experiments. #p >0.05, *p <0.05, **p <0.01, and ***p <0.001 (Student’s t-tests). **(E, F)** Cell lysates were subjected to Western blot analysis using antibodies against mtTFA and PGC-1α. β-actin was the protein loading control. The detailed sample order was lane 1: vehicle; lane 2 DSF; lane 3 CuCl; lane 4 CuCl_2_; lane 5 DSF with CuCl; lane 6 DSF with CuCl_2_. The protein bands were quantified through pixel density scanning and evaluated using ImageJ, version 1.44a (http://imagej.nih.gov/ij/) (accessed on 10 July 2024). The ratios of protein/β-actin were listed in the HEC-1-A and RL95–2 cells.

Based on the change in the mitochondrial fusion–fission cycle caused by the Cu^+^, Cu^2+^, DSF, and DSF–Cu^+^/Cu^2+^ complexes, we applied JC-1 dye to measure the mitochondrial membrane potential in HEC-1-A and RL95–2 cells (**Figure 6**). JC-1 dimers form in mitochondria with red fluorescence, and JC-1 monomers form in the cytoplasm with green fluorescence to measure mitochondrial depolarization, as indicated by an increase in green fluorescence intensity. Our JC-1 data showed that the DSF and DSF–Cu^+^/Cu^2+^ complexes increased the green fluorescence intensity in the HEC-1-A and RL95–2 cells, whereas Cu^+^ and Cu^2+^ decreased the green fluorescence intensity in the HEC-1-A and RL95–2 cells (**Figures 6A–D**). Mitochondrial transcription factor A (mtTFA) and peroxisome-proliferator-activated receptor γ co-activator-1α (PGC-1α) are two key mitochondrial respiratory and biogenic factors for mitochondrial respiratory functions (51, 52). Our Western blotting data showed that the Cu^+^, Cu^2+^, DSF, and DSF–Cu^+^/Cu^2+^ complexes decreased the mtTFA proteins in the RL95–2 cells and that the Cu^2+^, DSF, and DSF–Cu^+^/Cu^2+^ complexes decreased the PGC-1α proteins in the HEC-1-A cells (**Figures 6E, F**).

### 3.4 Effects of copper, DSF, and DSF–Cu^+^/Cu^2+^ complexes on proteins related to cell stresses in HEC-1-A and RL95–2 cells

**Figure 7:**
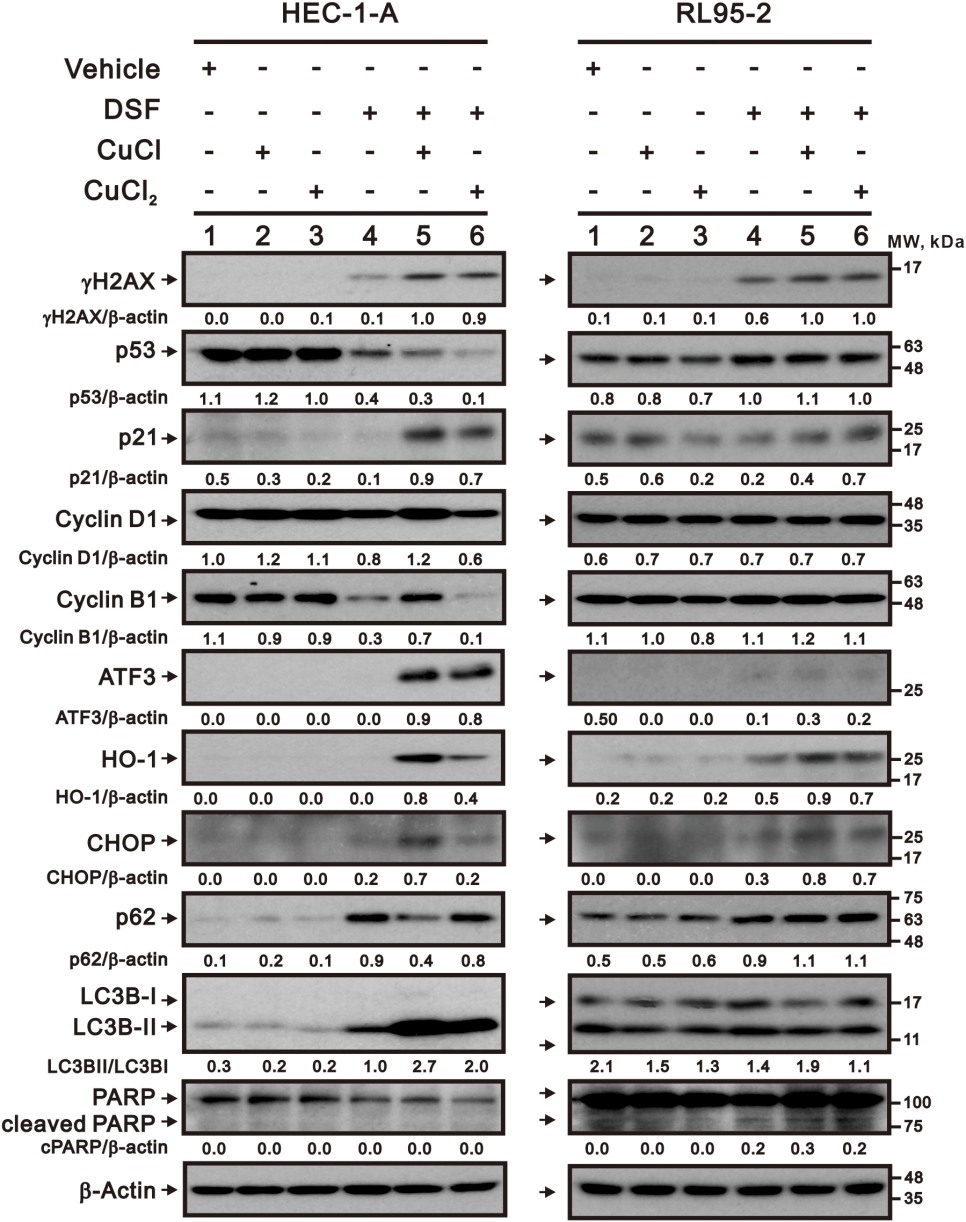
Effects of CuCl, CuCl_2_, and DSF on the cell cycle and cell stress-related proteins of human endometrial cancer cells. HEC-1-A (3 × 10^5^) cells were treated with 1 μM CuCl, 0.2 μM CuCl_2_, and 0.2 μM DSF, and RL95-2 (6 × 10^5^) cells were treated with 0.1 μM CuCl, 0.1 μM CuCl_2_, and 0.1 μM DSF for 48 h. The detailed sample order was lane 1: vehicle; lane 2 DSF; lane 3 CuCl; lane 4 CuCl_2_; lane 5 DSF with CuCl; lane 6 DSF with CuCl_2_. Cell lysates were subjected to Western blot analysis using antibodies against the indicated proteins. β-actin was the protein loading control. The protein bands from were quantified through pixel density scanning and evaluated using ImageJ, version 1.44a (http://imagej.nih.gov/ij/) (accessed on 10 July 2024). The ratios of protein/β-actin and LC3bII/LC3BI were listed in the HEC-1-A and RL95–2 cells.

We further examined cell cycle and cellular stress proteins using Western blotting analysis (**Figure 7**). We first examined proteins related to the cell cycle profile, including p53, p21, cyclin D1, and cyclin B1. Our data showed that p53 proteins were decreased by the DSF and DSF–Cu^+^/Cu^2+^ complexes; p21 proteins were increased by the DSF–Cu^+^/Cu^2+^ complexes; and cyclin D1 and cyclin B1 proteins were decreased by the DSF and DSF/Cu^2+^ complex in the HEC-1-A cells. In the RL95–2 cells, p53 proteins were increased by the DSF and DSF–Cu^+^/Cu^2+^ complexes, and p21 proteins were decreased by Cu^2+^, DSF, and the DSF/Cu^+^ complex. These cellular stress proteins were examined, including the DNA damage biomarker (γH2AX), oxidative stress biomarkers (ATF3 and HO-1), ER stress biomarker (CHOP), autophagy biomarkers (p62 and LC3BII), and apoptosis biomarker (cleaved PARP). In the HEC-1-A cells, γH2AX, CHOP, p62, and LC3BII proteins were increased by the DSF and the DSF–Cu^+^/Cu^2+^ complexes, and ATF3 and HO-1 proteins were increased by the DSF–Cu^+^/Cu^2+^ complexes. In the RL95–2 cells, the γH2AX, HO-1, CHOP, p62, and cleaved PARP proteins were increased by the DSF and DSF–Cu^+^/Cu^2+^ complexes.

### 3.5 The combination index of copper, DSF, and DSF–Cu^+^/Cu^2+^ complexes with cisplatin or doxorubicin in HEC-1-A and RL95–2 cells

**Figure 8:**
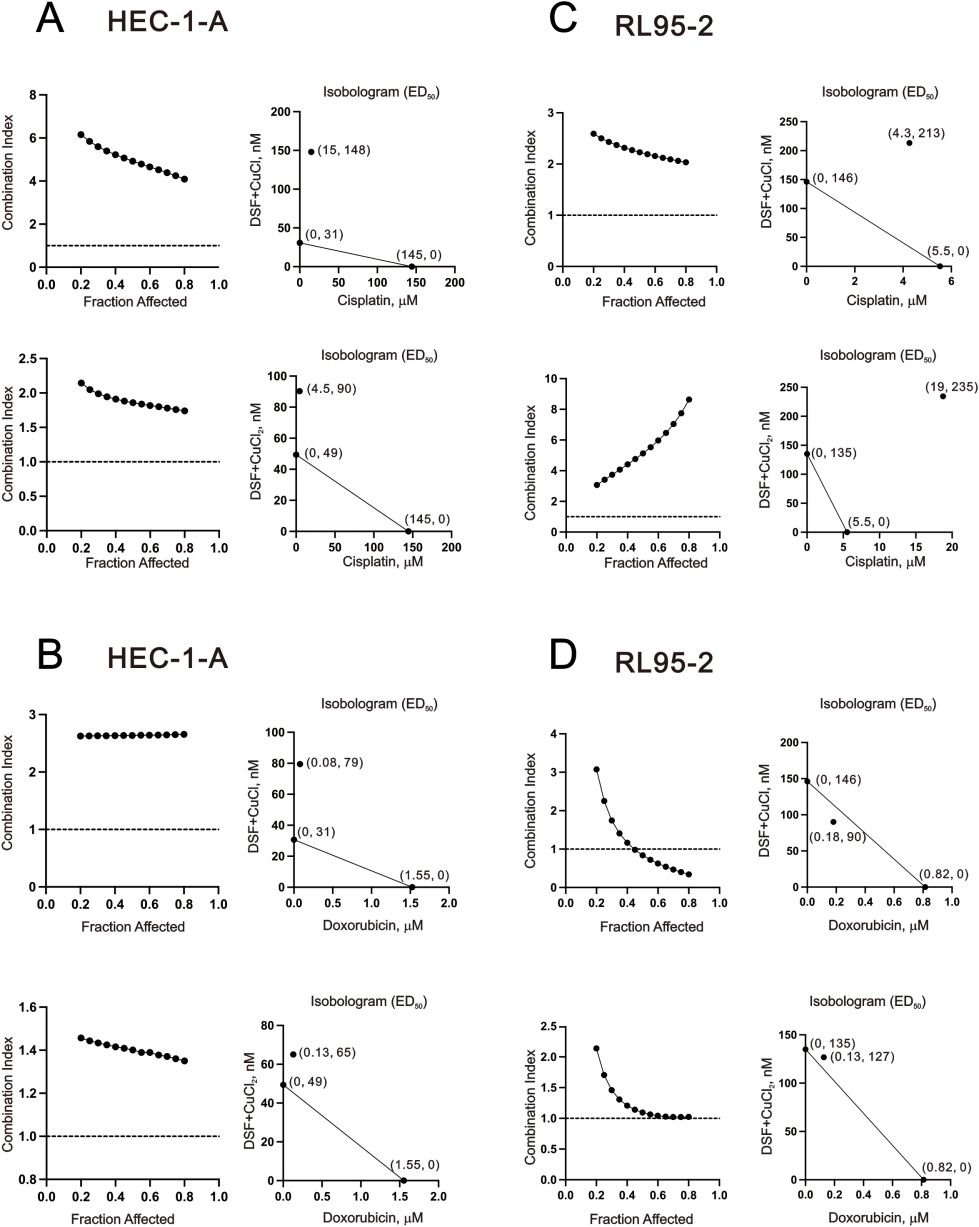
Combination index of DSF/CuCl and DSF/CuCl_2_ combined with cisplatin or doxorubicin in HEC-1-A and RL95–2 cells. **(A, B)** HEC-1-A (3 × 10^5^) cells were treated with cisplatin at doses of 0, 1.5625, 3.125, 6.25, 12.5, 25, 50, 100 µM or doxorubicin 0, 0.03125, 0.0625, 0.125, 0.25, 0.5, 1, 2 µM, combined with DSF/CuCl (1:1) or DSF/CuCl_2_ (1:1) at doses of 0, 0.003906, 0.007813, 0.015625, 0.03125, 0.0625, 0.125, 0.25, 0.5, and 1 μM. **(C, D)** RL95-2 (6 × 10^5^) cells were treated with cisplatin at doses of 0, 0.3125, 0.625, 1.25, 2.5, 5, 10, 20 µM or doxorubicin 0, 0.03125, 0.0625, 0.125, 0.25, 0.5, 1, 2 µM, combined with DSF/CuCl (1:1) or DSF/CuCl_2_ (1:1) at doses of 0, 0.003906, 0.007813, 0.015625, 0.03125, 0.0625, 0.125, 0.25, 0.5, and 1 μM. Cell viability was measured using the MTT method. The combination index and Isobolograms (ED_50_) were calculated using CalcuSyn software.

**Figure 9:**
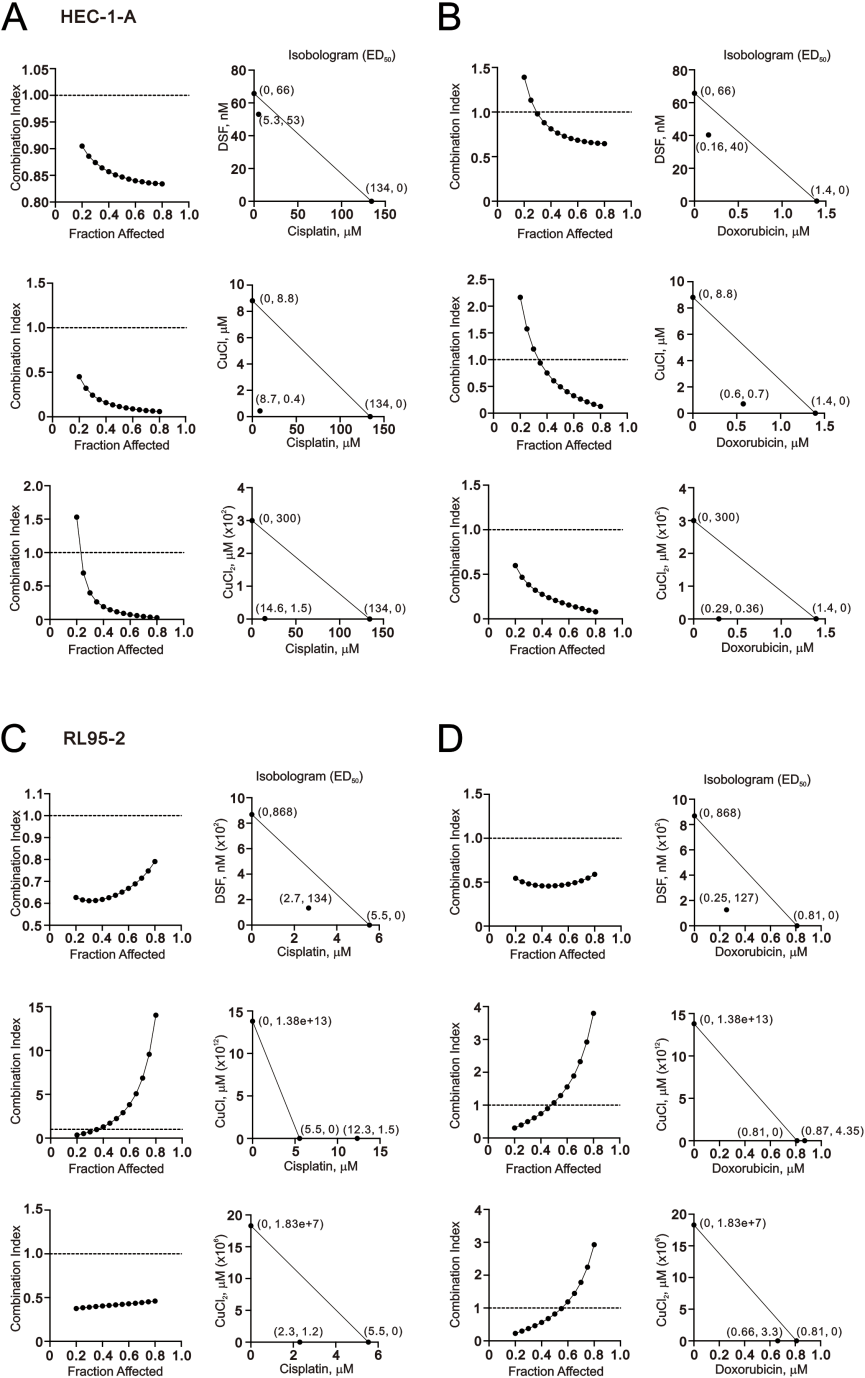
Combination index of CuCl, CuCl_2_, and DSF combined with cisplatin or doxorubicin in HEC-1-A and RL95–2 cells. **(A, B)** HEC-1-A (3 × 10^5^) cells were treated with DSF at doses of 0, 3.90625, 7.8125, 15.625, 31.25, 62.5, 125, 250, 500, and 1000 nM, CuCl dose: 0, 0.0.03906, 0.07813, 0.15625, 0.3125, 0.625, 1.25, 2.5, 5, and 10 μM, or CuCl_2_ dose: 0, 0.03906, 0.07813, 0.15625, 0.3125, 0.625, 1.25, 2.5, 5, and 10 μM combined with cisplatin dose: 0, 1.5625, 3.125, 6.25, 12.5, 25, 50, 100 µM or doxorubicin dose: 0, 0.0625, 0.125, 0.25, 0.5, 1, 2, and 4 µM for 24h. **(C, D)** RL95-2 (6 × 10^5^) cells were treated with DSF at doses of 0, 3.90625, 7.8125, 15.625, 31.25, 62.5, 125, 250, 500, and 1000 nM, CuCl dose: 0, 0.0.03906, 0.07813, 0.15625, 0.3125, 0.625, 1.25, 2.5, 5, and 10 μM, or CuCl_2_ dose: 0, 0.03906, 0.07813, 0.15625, 0.3125, 0.625, 1.25, 2.5, 5, and 10 μM combined with cisplatin dose: 0, 0.3125, 0.625, 1.25, 2.5, 5, 10, and 20 µM, or doxorubicin dose: 0, 0.03125, 0.0625, 0.125, 0.25, 0.5, 1, 2 µM for 24h. Cell viability was measured using the MTT method. The combination index and Isobolograms (ED_50_) were calculated using CalcuSyn software.

As presented in **Figure 1**, our study demonstrates the synergistic effect on the cytotoxicity of human endometrial cancer cells when DSF is combined with Cu^+^/Cu^2+^. Combination chemotherapy is proposed as a way to achieve synergistic effects and minimize drug doses, thereby enhancing treatment specificity for endometrial cancer patients and combating chemotherapeutic drug resistance (4). We conducted an examination to determine whether two commonly used chemotherapeutic drugs, cisplatin and doxorubicin, acted synergistically with DSF–Cu^+^/Cu^2+^ complexes in HEC-1-A and RL95–2 cells. Based on the data of **Figure 1**, we set the DSF/Cu^+^ (1:1) complex and DSF/Cu^2+^ (1:1) complex to measure whether they acted synergistically with cisplatin and doxorubicin in HEC-1-A and RL95–2 cells. Our combination index analysis showed that only the DSF/Cu^+^ complex acted synergistically with doxorubicin in the RL95–2 cells (**Figures 8A–D**). The CI values of the other combinations were above 1. By comparing **Figures 1**, **8**, we further examined the relationship between Cu^+^, Cu^2+^, or DSF and cisplatin and doxorubicin in HEC-1-A and RL95–2 cells. With the exception of the combination of Cu^+^ and cisplatin or doxorubicin in the RL95–2 cells, the combinations expressed the synergistic effects (**Figures 9A–D**). In the HEC-1-A cells, the ED50 values of cisplatin were reduced from 134 µM to 5.3 µM, 8.7 µM, and 14.6 µM by DSF, Cu^+^, and Cu^2+^, respectively; the ED50 values of doxorubicin were reduced from 1.4 µM to 0.16 µM, 0.6 µM, and 0.29 µM by DSF, Cu^+^, and Cu^2+^, respectively (**Figures 9A, B**). In the RL95–2 cells, the ED50 values of cisplatin were reduced from 5.5 µM to 2.7 µM and 2.3 µM by DSF and Cu^2+^, respectively; the ED50 values of doxorubicin were reduced from 0.81 µM to 0.25 µM and 0.66 µM by DSF and Cu^2+^, respectively (**Figures 9C, D**).

## 4 Discussion

Endometrial cancer primarily affects postmenopausal women, who often have comorbidities. The presence of comorbidities is associated with poorer survival among all endometrial cancer patients. Elevated levels of ROS have been demonstrated to trigger autophagy, apoptosis, and mitochondrial dysfunction in cells. We investigated whether DSF, copper ions, or DSF/Cu complexes exert antitumor effects through ROS production in two human endometrial carcinoma cell lines, HEC-1-A (type II) and RL95-2 (type I) cells. To develop effective treatments for endometrial cancer, it is essential to explore the diverse mechanisms through which DSF, CuCl, CuCl_2_, and DSF–Cu^+^/Cu^2+^ complexes, in combination with chemotherapy, can cause a decline in viability and induce cell death in endometrial cancer cells. In this study, our data showed that CuCl_2_, but not CuCl, acted synergistically with DSF to induce the cytotoxicity of HEC-1-A cells, and CuCl_2_ and CuCl both acted synergistically with DSF to induce the cytotoxicity of RL95–2 cells. The DSF–Cu^+^/Cu^2+^ complexes induced apoptosis, lipid peroxidation, autophagy, DNA damage, and ER stress in the HEC-1-A and RL95–2 cells. The DSF–Cu^+^/Cu^2+^ complexes elevated the amounts of cytosolic and mitochondrial ROS in the HEC-1-A cells, but not the RL95–2 cells. The DSF/Cu^+^ complex, but not the DSF/Cu^2+^ complex, resulted in mitochondrial depolarization in the HEC-1-A and RL95–2 cells. The combination of cisplatin or doxorubicin with the DSF–Cu^+^/Cu^2+^ complexes showed that only the DSF/Cu^+^ complex acted synergistically with doxorubicin in the RL95–2 cells. With the exception of CuCl combined with cisplatin or doxorubicin in the RL95–2 cells, DSF, CuCl, and CuCl_2_ each acted synergistically with cisplatin or doxorubicin in the HEC-1-A and RL95–2 cells. In summary, our current findings suggest that the differential effects of CuCl and CuCl_2_ complexed with DSF in the HEC-1-A and RL95–2 cells. In the RL95–2 cells, the entire valine codon 218 of p53 is deleted, while HEC-1-A cells, respectively, exhibit R248N point mutation (53). These differences might not be mediated through p53 transcriptional activity in endometrial cancer cells. It is a challenge to elucidate the status of copper ions and the context of endometrial cancer cells.

DSF has been shown to inhibit superoxide dismutase, compete with glutathione reductase, and block the ROS scavenging and detoxification mediated by ALDH isozymes. The increased ROS levels and inhibition of ROS scavenging mechanisms render the cells vulnerable to subsequent oxidative stress and further DNA damage, triggering DNA damage response mechanisms. The DSF/Cu complex induced replication stress-associated DNA damage in several cancer cell lines, leading to increased γH2AX levels; this serves as an indicator of DNA double-strand breaks in these cells. The activity of ALDH and the cancer stem cell phenotype have inspired scientists to develop specific ALDH inhibitors with greater clinical potential for the effective suppression of cancer stem cells (CSCs) and the inhibition of tumor progression (54). Additionally, DSF sensitized the cisplatin-resistant ALDH+ stem-like population in ovarian cancer to cisplatin treatment by suppressing ALDH activity and inducing apoptosis (55). Our previous study demonstrated that DSF and DSF/copper complexes failed to elevate the cytosolic and mitochondrial ROS levels independently of their inhibitor activity effect on ALDH enzyme activity in oral cells (46). Our current data showed that CuCl_2_ and DSF decreased the cytosolic and mitochondrial ROS levels and that the DSF–Cu^+^/Cu^2+^ complexes had no effect on these two ROS levels in the RL95–2 cells. Compared with the decline in the ROS level, DSF, CuCl, CuCl_2_, and the DSF–Cu^+^/Cu^2+^ complexes induced lipid peroxidation in the RL95–2 cells. The level of lipid peroxidation is directly related to the status of ROS in cells. The gap between lipid peroxidation and ROS should be clarified in RL95–2 cells. In addition, it should be a useful platform to explain the differential effect of DSF, CuCl, CuCl_2_, and DSF–Cu^+^/Cu^2+^ complexes.

The significant role of copper in facilitating cancer progression and the disruption of copper homeostasis in cancers have been extensively documented in both preclinical and clinical contexts (44). Higher levels of copper and oxidative stress are observed in cancer patients compared to healthy individuals. Copper-mediated oxidation leads to ROS production and subsequent cell death. Our previous study in human cervical cancer cells demonstrated the differential effects of copper (I) and copper (II) on the levels of HIF-1α, p53, p21, and the cPARP fragment, as well as the ability of DSF to counteract the effects of both copper (I) and copper (II). Our current data reveal the distinct functional roles of copper (I) and copper (II), as well as DSF/copper (I) and DSF/copper (II) in human endometrial cells. Studies have indicated that the DSF–Cu (exogenous copper II) complex serves as a more effective antineoplastic adjuvant than DSF alone. However, our current combination index analysis demonstrated that DSF–Cu^+^/Cu^2+^ complexes did not interact synergistically with cisplatin or doxorubicin in the HEC-1-A and RL95–2 cells. With the exception of CuCl in the RL95–2 cells, DSF, CuCl, and CuCl_2_ each exhibited synergistic effects with cisplatin or doxorubicin in both cell lines. Our current ED50 data also indicate that CuCl and CuCl_2_ alone did not exert apparent cytotoxicity in the HEC-1-A and RL95–2 cells. Therefore, it is crucial to verify the involvement of copper (I or II) in the Fenton reaction using Fenton reaction inhibitors and to confirm the true copper (I or II) status using copper (I or II) probes.

Our current ED_50_ values for cisplatin were consistent with the concept of the differential responsiveness of RL95–2 and HEC-1-A cells to cisplatin (56). However, we observed the cytotoxicity of DSF complexed with CuCl or CuCl_2_ and the synergistic effects between DSF, CuCl, and CuCl_2_, rather than the DSF–Cu^+^/Cu^2+^ complexes, with cisplatin or doxorubicin in the HEC-1-A and RL95–2 cells. The synergistic effects exerted by DSF, CuCl, or CuCl_2_ complexed with cisplatin or doxorubicin were more pronounced in the HEC-1-A cells than in the RL95–2 cells. These findings suggest numerous possibilities, including common and distinct pathways involving DSF, CuCl, and CuCl_2_ in human endometrial cancer cells. Considering the higher levels of copper in cancer patients compared to healthy subjects (44), it is plausible that chemotherapeutic drugs may perform better in patients with elevated copper levels. Our current findings may contribute to the addressal of the challenge posed by the lack of an efficacious chemotherapy regimen for the management of type II or recurrent endometrial cancer.

Building on our observation that DSF–Cu^+^/Cu^2+^ complexes trigger multi-layered stress responses (apoptosis, lipid peroxidation, autophagy, DNA damage, and ER stress) with cell-type–dependent redox phenotypes, including the discordance between ROS readouts and lipid peroxidation in RL95–2 cells and the distinct mitochondrial effects of DSF/Cu^+^ versus DSF/Cu^2+^, future studies should prioritize refining copper–redox interpretation and improving translational relevance. First, because “copper status” is a key uncertainty in endometrial cancer contexts, it will be essential to directly verify intracellular Cu^+^/Cu^2+^ speciation and Fenton-like redox engagement using copper-valence–selective probes and mechanistic inhibitors (e.g., Fenton reaction inhibitors), as we noted. Second, the unexpected pattern that DSF and copper salts (CuCl/CuCl_2_) frequently exhibited synergistic effects with cisplatin/doxorubicin, whereas pre-formed DSF–Cu^+^/Cu^2+^ complexes were largely non-synergistic (except DSF/Cu^+^ with doxorubicin in RL95–2 cells), motivates deeper investigation into copper speciation, complex stability/uptake kinetics, and schedule dependence to identify regimens that maximize efficacy while minimizing unnecessary copper-driven liabilities. Third, given that endometrial cancer predominantly affects postmenopausal women with comorbidities, where dose reduction and tolerability are clinically meaningful, future work should quantify the therapeutic window by testing DSF/Cu^+^/Cu^2+^ regimens in non-malignant endometrial epithelial and stromal models (and ideally patient-derived organoids/co-cultures) to calculate selectivity indices and guide safer dosing strategies.

In summary, our findings revealed that CuCl_2_, but not CuCl, interacted synergistically with DSF to induce cytotoxicity in HEC-1-A cells, whereas both CuCl_2_ and CuCl exhibited synergistic effects with DSF to induce cytotoxicity in RL95–2 cells. The DSF–Cu^+^/Cu^2+^ complexes induced apoptosis, lipid peroxidation, autophagy, DNA damage, and ER stress in both HEC-1-A and RL95–2 cells. Furthermore, the DSF–Cu^+^/Cu^2+^ complexes elevated cytosolic and mitochondrial ROS levels in HEC-1-A cells but not in RL95–2 cells. The DSF/Cu^+^ complex, rather than the DSF/Cu^2+^ complex, resulted in mitochondrial depolarization in both HEC-1-A and RL95–2 cells. When combined with cisplatin or doxorubicin, the DSF–Cu^+^/Cu^2+^ complexes showed that only the DSF/Cu^+^ complex interacted synergistically with doxorubicin in RL95–2 cells. With the exception of CuCl combined with cisplatin or doxorubicin in RL95–2 cells, DSF, CuCl, and CuCl_2_ each exhibited synergistic effects with cisplatin or doxorubicin in both HEC-1-A and RL95–2 cells. In summary, our current findings suggest distinct effects of CuCl and CuCl_2_ when complexed with DSF in HEC-1-A and RL95–2 cells. Future studies in relevant *in vivo* models will be important to validate these findings and further explore the translational potential of DSF–Cu complexes as anticancer therapeutics.

## Data Availability

The original contributions presented in the study are included in the article/supplementary material. Further inquiries can be directed to the corresponding author.
